# Diffuse and concentrated nitrogen sewage pollution in island environments with differing treatment systems

**DOI:** 10.1038/s41598-023-32105-6

**Published:** 2023-03-24

**Authors:** F. C. Alldred, D. R. Gröcke, C. Y. Leung, L. P. Wright, N. Banfield

**Affiliations:** 1 Department of Earth Sciences, University of Durham, South Road, Durham, County Durham DH1 3LE UK; 2 Isles of Scilly Wildlife Trust, Trenoweth, St Mary’s, Isles of Scilly TR21 0NS UK; 3 Present Address: Buglife-The Invertebrate Conservation Trust, G.06, Allia Future Business Centre, London Road, Peterborough, PE2 8AN UK

**Keywords:** Geochemistry, Marine chemistry

## Abstract

Macroalgae is an under-utilised tool as a bioindicator of anthropogenic nitrogen loading to the coastal environment in the UK. This study compared two island systems—Jersey (Channel Islands) and St Mary’s (Isles of Scilly) to assess how differing sewerage infrastructure affects nitrogen loading. A total of 831 macroalgae samples of *Fucus vesiculosus* and *Ulva* sp. were analysed for nitrogen isotopes (δ^15^N). Elevated δ^15^N values were recorded for Jersey (> 9‰) in St Aubin’s Bay—caused by the outflow of the Bellozanne Sewerage Treatment Works (STW). δ^15^N isoplots maps indicate low diffusion of nitrogen out of St Aubin’s Bay. St Mary’s produced a varied δ^15^N isoplot map in comparison. δ^15^N was typically lower and is attributed to a smaller population and inefficient STW. Outflow of sewage/effluent at Morning Point, Hugh Town and Old Town produced elevated δ^15^N values in comparison to the island average. St Mary’s inefficient sewerage treatment and reliance on septic tanks/soakaways complicates δ^15^N interpretation although it still indicates that nitrogen pollution is an island-wide issue. Future sewerage development and upgrades on islands are required to prevent similar effluent environmental issues as recorded in St Aubin’s Bay. This study advocates the use of macroalgae as a bioindicator of nitrogen effluent in the marine environment.

## Introduction

Anthropogenic activity has altered the modern nitrogen cycle primarily through the increased influx of sewage to marine environments^1^. It has been estimated that biologically available nitrogen to the ocean has doubled in rate between 1960 and 1990 and hence, is no longer a limiting factor on marine ecosystem productivity^2–4^. Sewage outfalls have been linked to multiple instances of eutrophication, whereby enhanced nutrient uptake by algae results in blooms and hypoxia in the marine environment^5^. Monitoring sewage pollution extent, severity and point source is critical to minimise the risk to public and environmental health^1,5^.

Traditional nitrogen isotope monitoring techniques rely on analysing the dissolved inorganic nitrogen content of seawater, which is time-consuming and expensive in comparison to nitrogen isotope analysis of macroalgae^6^. Nitrogen isotope ratios (expressed as δ^15^N) from macroalgae have been shown to accurately record the nitrogen isotope composition of seawater^1,7^. In fact, nitrogen uptake in macroalgae has successfully traced the influence of sewage/effluent up to 24 km from a point source^3^. The ability to distinguish nitrogen pollution sources come from the premise that sewage/effluent δ^15^N exhibits more elevated values compared to chemical industry sources^7,8^. This is primarily due to physical, chemical, and biological processes (denitrification^9^) that occur during the treatment of sewage at wastewater treatment plants. The process of denitrification preferentially removes ^14^N from the effluent, hence producing a discharge with more positive δ^15^N values > + 8‰^3,7,10^. When sewage/effluent is released without any denitrification processes the δ^15^N will reflect the source (e.g., artificial fertiliser, animal/human sewage; see below for discussion)^8^. Typically, coastal environments that are affected by sewage range between + 4‰ to + 19‰^8,11^.

Savage and Elmgren^12^ recommend that values below ~ + 4 ‰ reflect artificially produced nitrate that uses atmospheric nitrogen (~ 0‰) as its nitrogen source. Most agricultural fertilisers use artificially produced products and thus, typically exhibit low or negative δ^15^N values^7^. Therefore, macroalgae in a coastal environment dominantly affected through fertilizer runoff will exhibit near zero or negative δ^15^N values. When animal and/or human sewage/effluent reaches the coastal environment unprocessed (i.e., raw) or processed (denitrification), the δ^15^N value of coastal macroalgae will be elevated (since herbivore/human δ^15^N values for the British Isles range between + 4‰ to + 8‰^13^) in comparison to artificial fertilizers and/or background marine signatures (e.g., artificial fertilizers < + 4‰, unpolluted/background + 4‰ to + 6‰, sewage/effluent/manure polluted = > + 6‰). However, coastal and/or estuarine environments influenced by wastewater treatment plants will exhibit much higher δ^15^N values (e.g., > + 10‰^3^) in comparison to untreated sewage (e.g., > + 6‰). Large-scale, coastal macroalgae δ^15^N isoplot maps will help differentiate and determine point sources of pollution, which can then influence societal practices and policies in that region^14^.

The macroalgae, *Fucus vesiculosus* (bladder wrack) and *Ulva* sp. are commonly used in nitrogen isotope studies of the coastal environment^15,16^. *Ulva* sp. are more opportunistic and will bloom when concentrations of nitrogen in the water column become elevated^17^. Both macroalgae are found around the coastlines of Europe, with *F. vesiculosus* commonly found in the intertidal zone of rocky shores, while *Ulva* sp. prefers sheltered habitats^18,19^. Dissolved inorganic nitrogen is incorporated within 13–19 days in *F. vesiculosus*, whereas in *Ulva* sp. it has been reported to be within as little as 48 h^6,11,16,20^. Although the use of nitrogen isotopes in macroalgae is cheaper and quicker, it is not widely used^15^ and there are only a handful of studies on UK coastlines^6,11,15^. This is surprising considering the exponential increase of unregulated release of sewage/effluent into UK rivers since leaving the European Union (i.e., Brexit)^21^.

In this study, we performed nitrogen isotope analysis of *F. vesiculosus* and *Ulva* sp. (simply referred to as *Fucus* and *Ulva* hereafter) from two contrasting island environments in terms of wastewater treatment processes: Jersey has a centralised wastewater treatment facility that is currently being upgraded, and St Mary’s (Isles of Scilly) has no major wastewater treatment facility but relies on continuous outflow, septic tanks and soakaway systems.

## Study sites

**Figure 1:**
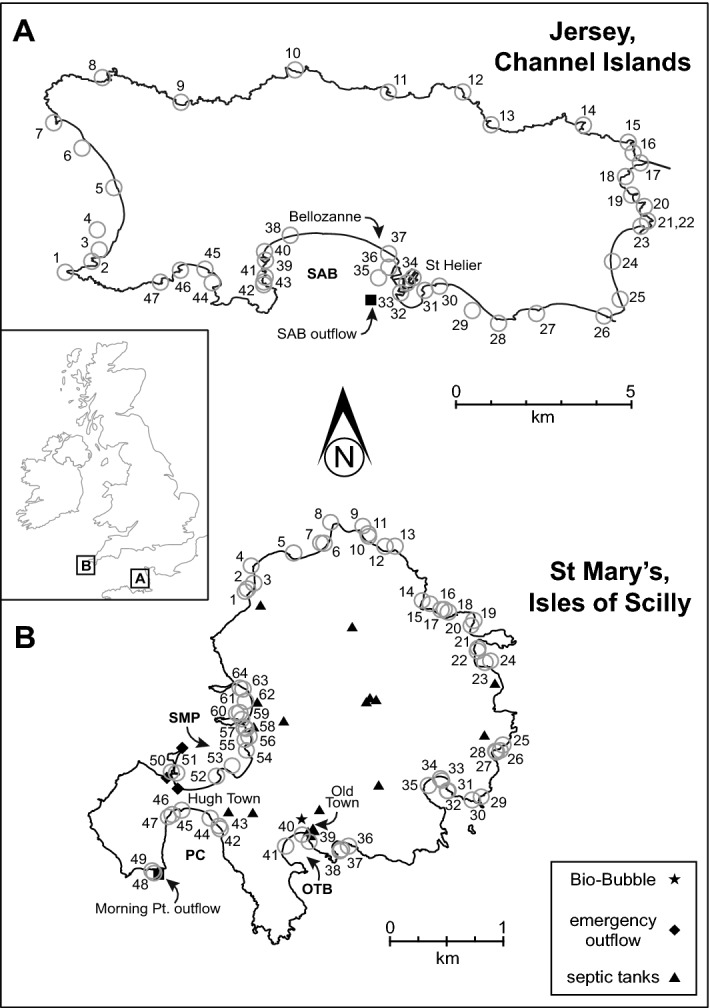
Map of the islands investigated in this study: (**A**) Jersey, Channel Islands; (**B**) St Mary’s, Isles of Scilly. Sample locations are indicated by numbered grey circles. Key locations discussed in text are also indicated. *SAB* St Aubin’s Bay, *SMP* St Mary’s Pool, *PC* Porth Cressa, *OTB* Old Town Bay. Note different scales for each island.

Jersey is the largest of the Channel Islands located 14 miles off the coast of Normandy, France. As of 2019 the population was 106,800 increasing by ~ 1200 persons per year, and the majority of the population resides in St Helier and St Aubin’s Bay (Fig. 1A)^22^. Jersey relies solely on the Bellozanne Sewerage Treatment Work (STW) (Fig. 1A) for all sewage treatment on the island. Prior to its commission in 1959, untreated sewage was discharged directly into the sea. The STW was built for a population of 57,000 and so continuous improvements have been required as the population has grown and environmental standards have changed^23^. Despite continued improvements, surveys have shown that St Aubin’s Bay continues to exhibit high levels of nutrient loading from effluent^24,25^. Trophic status reports carried out by the Centre for Research into Environmental Health (CREH) in 1997 suggest winter hyper-nutrification in St Aubin’s Bay; despite the predicted 2% to 21% reduction in chlorophyll concentration from nutrient removal by the Bellozanne STW^25^. A 2010 reassessment shows considerable reduction in nitrogen load compared to 1997 values, and modelling indicates no potential eutrophication in the bay^26^. However, the STW has continued to fail the Total Nitrogen limit set at 10 mg/l, instead recording between 11 and 63 mg/l with an average of 31.3 mg/l between 2009 and 2015^27,28^. No further upgrades are possible for the Bellozanne STW and it cannot support the Jersey population as it currently stands. Sewage effluent entering the bay is causing increased nitrogen loading that is resulting in substantial *Ulva* growth and eutrophication in St Aubin’s Bay^27,29^; the Jersey Government report that *Ulva* growth in St Aubin’s Bay will always persist^29^. The construction of a new STW at Bellozanne began in 2018 and will be operational in 2023 and aims to reduce discharge of partially treated sewage by 97%^30^. The new STW will support a population size of 118,000 and aims to also reduce nitrogen loading to St Aubin’s Bay by an additional 10–15% compared to the current STW outflow.

The Isles of Scilly (IOS) are a group of islands situated 27 miles southwest off the coast of the UK (Fig. 1B). The islands sit at the end of the North Atlantic Current and the Gulf Stream producing a milder climate compared to mainland UK, with winter surface temperatures of ~ 10 °C^31^. Two tidal jets transport water around the isles, the primary operates in a clockwise direction transporting water south, the secondary current transports water northwards^32^. St Mary’s has an area of 6 square miles and is the largest island in the IOS. The permanent population is ~ 1800, although this increases to > 6000 in summer months^33^. Most of the island’s population lives in the southwest in Hugh Town (Fig. 1B). The northwest supports small-scale agriculture (6–7 hectares) with no intensive agriculture, and as such, the coastal waters are generally classified as pristine^34^. There is no wastewater treatment facility on St Mary’s—instead the island relies on several smaller wastewater infrastructures. There is a 3 km sewerage network in Hugh Town supporting ~ 800 properties and connects to the Morning Point outflow (Fig. 1B). Morning Point is the only permitted outflow on the IOS where untreated raw sewage is discharged into the Atlantic^35^. Two emergency outflows intermittently discharge sewage into the Hugh Town harbour in the case of flood events (Fig. 1B)^35^. Old Town is serviced by a combination of a Bio-Bubble facility (i.e., an advanced aeration system, https://www.bio-bubble.com) connected a soak-away draining to Old Town Bay, the Morning Point network, and three septic tanks^33,35^. Septic tanks serve as the main sewage treatment process for properties outside of Hugh Town and Old Town. The Environment Agency does not provide a full list of all septic tanks on the island; thus, it must be noted that more septic tanks than those presented in Fig. 1B are likely to be in operation^36^.

The private company South West Water (SWW) has taken over responsibility for sewage treatment on the IOS from the local council. SWW aim to reduce sewage pollution by 2030 and and report that St Mary’s sewerage network is in need of major repair^35,37^. Poor record keeping by the IOS council has resulted in a lack of information regarding sewerage leaks and septic tank conditions across the island^35^. By 2030, SWW aims to build a resilient treatment facility and upgrade the current Bio-Bubble^37^. Initial plans by SWW^35^ considered closing the Morning Point outflow, with discharge redirected to the emergency outflow into St Mary’s Pool (Fig. 1B). The emergency outflow pipeline would be upgraded, and households encouraged to connect to the sewerage network and thus, reducing the reliance on septic tanks^37^.

## Materials and methods

Macroalgae from Jersey was collected during December 2020 and January 2021 by one of us (LPW) during the Covid pandemic. 372 samples were collected from 47 sites around Jersey (Fig. 1A) consisting of 336 *Fucus* and 36 *Ulva* samples. Macroalgae from the IOS was collected by one of us (NB) during the same time period. 459 macroalgae samples were collected from 62 sites (Fig. 1B) consisting of 429 *Fucus* and 30 *Ulva* samples. Sample sites from both locations were selected primarily on the presence of macroalgae and ease of accessibility for collecting. The outermost non-fertile tip of *Fucus* was sampled from several different specimens, whereas a 4 cm-square area of *Ulva* was sampled and compressed to remove all seawater. Each sample was placed in a small brown envelope, labelled and subsequently dried in their envelopes soon after collection in a domestic oven set between 45 and 60 °C. Sub-samples of compressed *Ulva* and the margin of the non-fertile tip of *Fucus* were weighed into tin capsules for subsequent stable isotope analysis following the protocols outlined in Gröcke et al.^6^ and Bailes and Gröcke^11^. QGIS software was used to produce the isoplot maps of δ^15^N around Jersey and St Mary’s.

## Results

**Figure 2:**
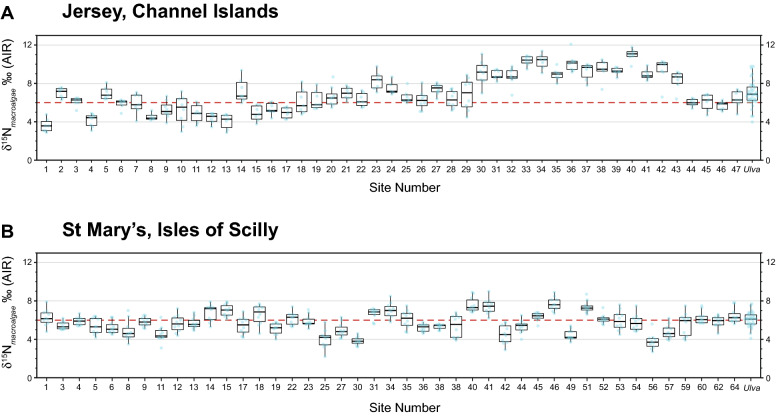
Box and whiskers plots of macroalgae δ^15^N from Jersey (**A**) and St Mary’s (**B**). All numbered sites represent *Fucus* results. All *Ulva* results for the islands have been grouped as one dataset and are presented on the farthest right of each graph. δ^15^N values above the dashed red line represent coastal environments that are affected by sewage/effluent. Macroalgae δ^15^N elevation is recorded for St Aubin’s Bay (Sites 30–43) linked to the Bellozanne STW outflow.

The average δ^15^N value from Jersey for *Fucus* is + 7.0‰ ± 1.9‰ (*n* = 336) and + 7.1‰ ± 1.3‰ (*n* = 36) for *Ulva*, which are above natural background levels reported between + 4‰ and + 6‰^8,12^. δ^15^N values of *Fucus* range between + 2.9‰ (Sites 1 and 13) and + 12.1‰ (Site 36) showing elevated δ^15^N in St Aubin’s Bay for both *Fucus* and *Ulva* (Fig. 2A). Site 40 recorded the greatest elevation in δ^15^N for *Fucus* (+ 11.0‰ ± 0.6‰, *n* = 7) and the five most elevated δ^15^N (> + 9.0‰) sites (33, 34, 36, 38 and 40) are found in St Aubin’s Bay. *Ulva* recorded a δ^15^N range between + 4.3‰ and + 9.8‰ (Fig. 2A). Lower average δ^15^N values were recorded in *Fucus* for the north coastline (+ 5.1‰ ± 1.3‰, *n* = 57), the west coastline (+ 5.7‰ ± 1.4‰, *n* = 78) and for the east coastline (+ 6.5‰ ± 1.2‰, *n* = 69) in comparison to the southern coastline (+ 8.3‰ ± 1.8‰, *n* = 160). Although there are less data for *Ulva* it recorded higher δ^15^N average values for each coastline compared to *Fucus* for the north, east and west coastlines (+ 5.6‰ ± 1.1‰, *n* = 7; + 6.7‰ ± 0.5‰, *n* = 10; and + 7.1‰ ± 0.6‰, *n* = 6, respectively). However, the south coast showed a depleted δ^15^N average (+ 7.8‰ ± 1.4‰, *n* = 14) compared to *Fucus*. Macroalgae in St Aubin’s Bay produced a significantly more elevated δ^15^N signature in comparison to the whole island average; this is observed in both *Fucus* (+ 9.5‰ ± 1.1‰, *n* = 85) and *Ulva* sp. (+ 8.3‰ ± 1.2‰, *n* = 8) (with a *p* value < 0.05; see Supplementary Fig. 1).

From the IOS, *Fucus* recorded an average δ^15^N value of + 5.8‰ ± 1.2‰ (*n* = 429), and *Ulva* recorded an average δ^15^N value of + 6.2‰ ± 0.7‰ (*n* = 30). IOS seagrass has an average δ^15^N value of ~ + 5‰ which is on the low side of our dataset^38^. *Fucus* ranged between + 2.2‰ (Site 25) and + 9.0‰ (Site 41), whereas *Ulva* varied between + 4.1‰ and + 7.7‰ (Fig. 2B). δ^15^N values are slightly skewed towards more elevated δ^15^N values along the southern (+ 5.9‰ ± 1.4‰, *n* = 99), western (+ 5.8‰ ± 1.2‰, *n* = 99) and northern coastlines (+ 5.8‰ ± 1.0‰, *n* = 140) compared to the eastern coastline (+ 5.5‰ ± 1.3‰, *n* = 90). Site 46 recorded the most elevated average δ^15^N value from the IOS (+ 7.7‰ ± 0.7‰, *n* = 10), which is located at Porth Cressa. The three other most elevated δ^15^N (> 7.0‰) sites are also in the southwest of the island (Sites 40, 41, 51) (Fig. 2B). Average δ^15^N values decrease in distance away from Morning Point (see Supplementary Fig. 2). No correlation exists between site average δ^15^N values and proximity to septic tanks (see Supplementary Figs. 3 and 4). Sites 30 and 56 recorded the lowest δ^15^N average value for *Fucus* (+ 3.8‰ ± 0.4‰, *n* = 10 and + 3.8‰ ± 0.8‰, *n* = 10, respectively). *Ulva* was found to have the lowest δ^15^N values along the east coastline (+ 5.6‰ ± 0.4‰), which is in line with results from *Fucus*.

## Discussion

### Jersey: focused point-source effluent pollution

**Figure 3:**
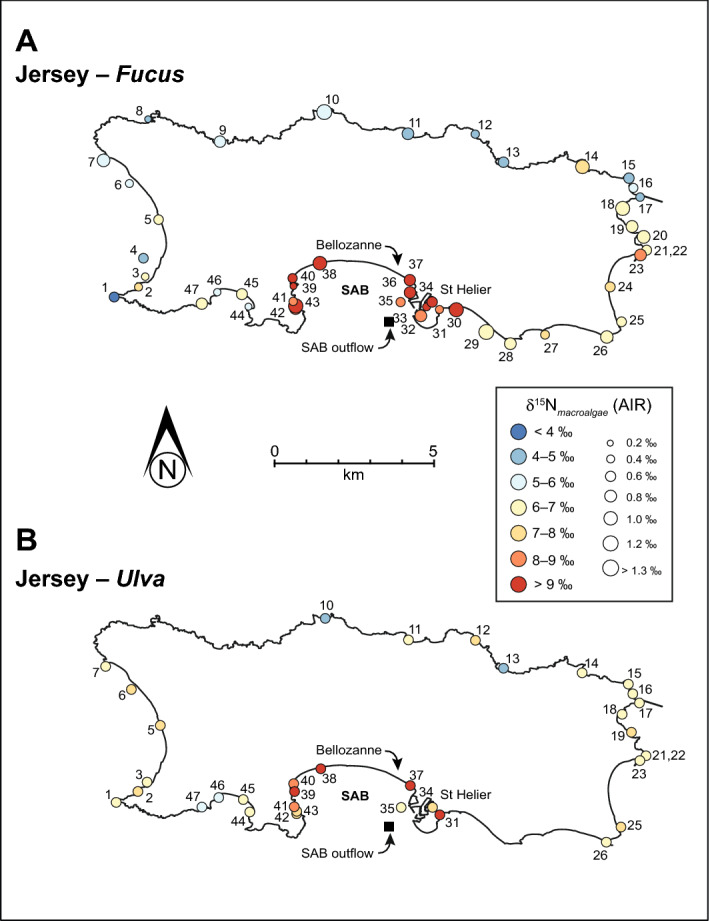
δ^15^N isoplot map for the macroalgae, *Fucus* (**A**) and *Ulva* (**B**) around Jersey, Channel Islands. Colour range represent distinct δ^15^N value ranges, while the size of the circle represents the standard deviation of δ^15^N from each site. Elevated macroalgae δ^15^N values are recorded for St Aubin’s Bay (Sites 30–43) and is associated to the Bellozanne STW outflow. Normal background macroalgae δ^15^N values are located on the west and north coastlines (blue circles). For abbreviations refer to Fig. 1.

The δ^15^N values from macroalgae around the Jersey coast demonstrate a clear geospatial pattern with more elevated δ^15^N values in St Aubin’s Bay compared to the remainder of the island (Fig. 3A). Multiple elevated δ^15^N values > + 6.0‰ indicate nitrogen loading from an anthropogenic effluent source^1,12,39^. The position of the Bellozanne STW corresponds with these elevated δ^15^N sites (Fig. 3A for *Fucus* and Fig. 3B for *Ulva*). Such elevated δ^15^N values are in line with previous studies of macroalgae near STW^1,39^. δ^15^N of groundwater sampled from Jersey in 1995 also show elevated values (+ 11.8‰ to + 18.4‰ in the St Aubin’s Bay coastal margin^40^. These elevated δ^15^N values correspond to the lowest nitrate concentrations in the groundwater and are interpreted as a result of denitrification processes in the deep groundwater system. Quantitative information on the mixing zone between groundwater and seawater is not available for St Aubin’s Bay and thus, we are uncertain of the influence of deep groundwater δ^15^N on macroalgae δ^15^N. Although data on Total Dissolved Solids for Jersey indicate that saline intrusion occurs around St Aubin’s Bay and other low-lying regions, and thus would imply little influence from deep groundwater^41^.

The lack of elevated δ^15^N values elsewhere around Jersey strongly suggests that the release of sewage effluent nitrogen in St Aubin’s Bay is not transported around the island through oceanic currents. St Aubin’s Bay has a large tidal range (on the order of 10 m) and is sheltered by the westerlies and Atlantic swell, thus causing reduced mixing with the open ocean. Limited water exchange with the open ocean reduces the capacity for effluent diffusion as well as reducing δ^15^N via ammonia volatisation^3,42,43^. Macroalgae δ^15^N data from Jersey shows that the geographic position of STWs and the placement of outflow pipes require a thorough environmental assessment, considering—at least—prevailing wind and oceanic and tidal currents prior to any planning and development. If such investigations were conducted in Jersey, St Aubin’s Bay may not be the environmental disaster it has become^44,45^. The north, east and west coastlines of Jersey reflect δ^15^N values typical of natural background levels, suggesting minimal anthropogenic nitrogen sources (effluent, fertilizer, industrial sources) (Fig. 3A) and/or that oceanic currents, tidal ranges and bay openness are dissipating any nitrogen loading into the open ocean.

### St Mary’s: dispersed point-source effluent pollution

**Figure 4:**
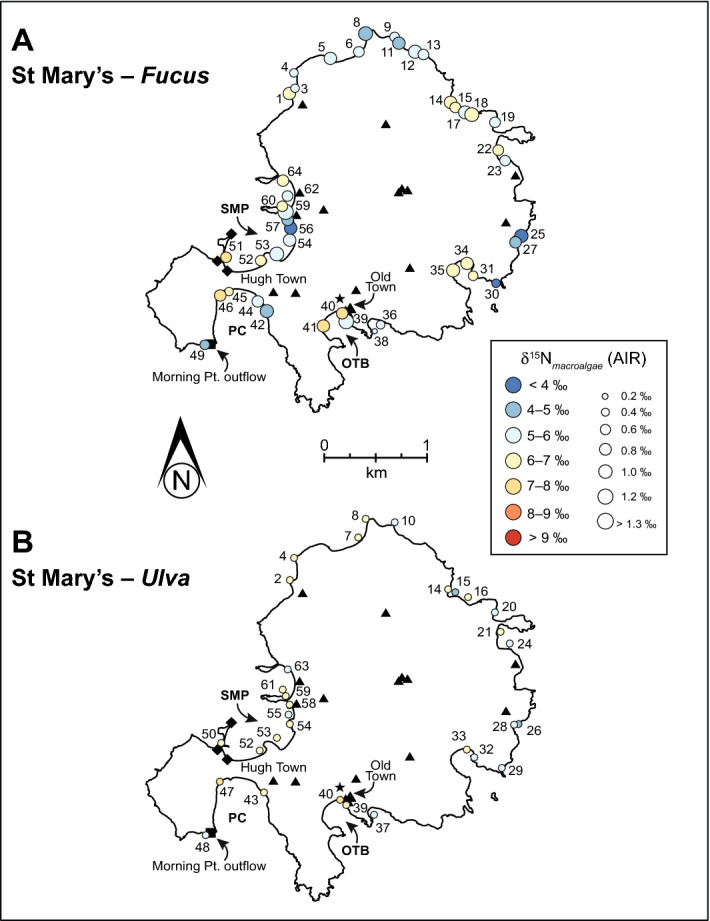
δ^15^N isoplot map for the macroalgae, *Fucus* (**A**) and *Ulva* (**B**) around St Mary’s, Isles of Scilly. Colour range represent distinct δ^15^N value ranges, while the size of the circle represents the standard deviation of δ^15^N from each site. In comparison to Jersey, macroalgae δ^15^N values are not as elevated (e.g., with dark orange or red circles) on St Mary’s: most likely related to the lower population size, release of untreated sewage/effluent and/or the islands reliance on a septic tank/soakaway system. Elevated macroalgae δ^15^N values are recorded for Morning Point, Hugh Town and Old Town regions. For abbreviations refer to Fig. 1.

In comparison to Jersey, St Mary’s in the IOS exhibits more complex nitrogen loading and geospatial variation in δ^15^N around the coastline. The main difference causing this complexity is the difference in sewerage treatment approaches between the islands: a single, principal sewage treatment plant *versus* a spatial array of septic tanks and two isolated sewerage systems in disrepair around the island. Several sites around St Mary’s coastline record elevated δ^15^N values in comparison to normal, healthy marine values: Sites 41, 46 and 51 are located in three separate bays (Fig. 4A). Nitrogen pollution point sources on St Mary’s can be separated into three main infrastructures: Morning Point discharge, septic tanks/soakaways and the Hugh Town emergency outflow. Figure 4A reveals that the two most elevated δ^15^N sites correspond to Morning Point discharge and the Hugh Town emergency outflow. Site 46 has the most elevated δ^15^N values on the island and is the closest sampled point to the sewage outflow at Morning Point, discharging untreated and semi-treated sewage into Porth Cressa (Fig. 4A,B)^33^. A steady decline in δ^15^N values is observed across a 160 m profile at Porth Cressa, indicating diffusion of nitrogen loading from the Morning Point source into the bay: even with limited data this pattern is also reflected in *Ulva* (see Supplementary Fig. 2). A lowering of macroalgae δ^15^N away from a source outflow would also suggest that Porth Cressa is not well-mixed, allowing the opportunity for the effluent to be absorbed by the local environment (e.g., macroalgae, microalgae, microflora, sediment). The relationship between decreasing δ^15^N and distance from a nitrogen source has been recorded in other macroalgae investigations^1,12,20^.

A significant proportion of the population (residential and tourist) on St Mary’s rely on septic tanks and soakaways as a method for sewage processing, storage and release. As a result of sewage storage in septic tanks, the septic tank environment would start with nitrification processes (e.g., ammonia oxidisation to nitrate) in an oxygenated empty tank and then shift to anaerobic denitrification once oxygen becomes depleted; at that point denitrification would further elevate the δ^15^N signature of the sludge and effluent^46,47^. Reduced regulation and monitoring of septic tanks will lead to their disrepair, allowing them to leak nutrients (nitrogen and phosphorus) into the groundwater system and eventually to the coastal environment.

Old Town has a soakaway delivering effluent from the Bio-Bubble to Old Town Bay. Although this effluent may have slightly elevated δ^15^N (e.g., animal/human signature^13^), the aeration process (i.e., nitrification processes) would not elevate it further. Site 41 at Old Town Bay has the highest individual δ^15^N value (+ 9.0‰) for the entire island. Therefore, elevated δ^15^N at Old Town Bay may be attributed instead to the three septic tanks located nearby (see Fig. 4). Sites around St Mary’s that are located near septic tanks exhibit large standard deviations (> 0.7‰), but there is no trend with distance from the source (Sites 39–41, 56–64) (see Supplementary Figs. 3 and 4). Although legislation on St Mary’s does not allow for the discharge of effluent from septic tanks the maintenance of these tanks is not regulated: septic tanks are meant to be emptied and the contents transported to the mainland of England for processing^33,48^—although it is known by one of us (NB) that emptied tanks are not being transported back to the mainland. Farmers may be managing their own septic tank systems, such as those on Bryher, IOS^21^. In addition, many septic tanks on the island have soak-aways that may have some degree of contact with oxygen causing nitrification processes to occur (e.g., lowering δ^15^N values)^12^. Elevated δ^15^N values recorded at the above-named sites may be caused from poorly maintained septic tank systems or from groundwater denitrification processes at depth producing less distinct source point pollution areas with elevated δ^15^N signatures^40,47,48^.

Another issue that complicates the interpretation of macroalgae δ^15^N around St Mary’s is the geographic position of the island and surrounding islands, the structure of the coastline and ocean circulation/currents. Viana and Bode^43^ showed that open coastal waters along the coast of Spain recorded lower δ^15^N values in macroalgae compared to enclosed bays. For example, elevated δ^15^N values at Sites 31–35 for *Fucus* and *Ulva* are potentially caused by the shape of the bay restricting water movement, causing ^15^N enrichment. There are no obvious sewage/effluent sources to the eastern coastline. A direct comparison between our macroalgae δ^15^N data with IOS seagrass δ^15^N data^38^ is not possible since latitudinal information was not provided for the seagrass data. However, the seagrass δ^15^N data is typically < + 5‰, suggesting that the nitrogen isotope signature of effluent is not reaching the seagrass community further out from the coastline. Sewage nitrogen trapped in bays has a longer time to become isotopically fractionated, and therefore could lead to an elevated δ^15^N signature that is subsequently incorporated into macroalgae^3^. The enclosed nature of Porth Cressa, may also be preserving and/or enhancing the elevated δ^15^N values in macroalgae at this site. Another complicating factor is the clockwise movement of oceanic water around the IOS, which has the potential for transporting effluent-contaminated waters away from the north-west coastline producing less elevated macroalgae δ^15^N values on the isoplot map^32^.

### To treat or not to treat: effluent pollution in island environments

As shown in this study, a single, operational wastewater treatment plant for an entire island has the potential to concentrate effluent release to a single point/area, resulting in elevated coastal macroalgae δ^15^N values. The principal issue in Jersey is that the outflow of the Bellazonne STW is directed solely into St Aubin’s Bay. This causes elevated nitrogen loading in the bay which has a direct impact on the health of the ecosystem. The nature of the oceanic conditions in the region and the fact that coastal bays are natural traps means that nitrogen loading is intensified, causing significant environmental issues—for example, green tides, high levels of nitrogenous compounds and obvious water discolouration^44,45,49,50^. In direct contrast, St Mary’s has no centralised wastewater treatment plant and therefore effluent is either/or; (1) directly released into the ocean with no and/or limited treatment, and unregulated emptying of septic tanks; and/or (2) an antiquated, sewerage system in need of significant investment to upgrade it. The lack of adequate wastewater treatment on St Mary’s may be the cause behind lower macroalgae δ^15^N values compared to Jersey. Although the δ^15^N values may be lower, indicating less denitrification, this does not equate to less nitrogen loading in the coastal environment around St Mary’s. In fact, groundwater nitrate concentrations from 1995 on St Mary’s averaged 58 mg/l^51^, which exceeds the World Health Organisation guideline value: no current nitrate or Total Nitrogen is publicly available for St Mary’s. Irrespectively, nitrogen chemical analyses (i.e., nitrogen pollution) would need to be monitored around the entire island of St Mary’s, compared to Jersey which could focus on the one region (i.e., St Aubin’s Bay), to monitor effluent contamination. Therefore, despite the significantly smaller population size on St Mary’s nitrogen loading may equally be having an impact on the coastal environment and ecosystems (e.g., on eelgrass, *Zostera marina*, populations and genetic diversity around the IOS^52^).

In the future, wastewater treatment plants require more thorough development and planning on islands, specifically in terms of a more comprehensive environmental assessment that considers oceanic circulation, modelling the effect of swell, geography and bathymetry of the coastline, tidal ranges, and any other ocean–atmosphere environmental conditions; this must all occur prior to placement of an outflow pipe. St Aubin’s Bay clearly demonstrates a lack of foresight when positioning the outflow, since it is now causing environmental issues in that bay all-year round^27–29,44,45^. The Morning Point outflow on St Mary’s is also positioned incorrectly, as evidenced by the elevated δ^15^N values at Porth Cressa: this beach also encounters problems associated with abundant macroalgae and *Ulva* sp. reflecting nitrogen-rich waters. A more confusing geospatial pattern in macroalgae δ^15^N values around St Mary’s is thought to be caused by the island’s reliance on managed and unregulated septic tanks, and an inadequate sewerage system.

## Conclusions

Nitrogen isotopes of macroalgae around island coastlines can offer valuable insight into sewerage practices and effluent pollution affecting the region. This study shows that Jersey, which relies on an inadequate STW facility is unable to cope with current and projected population demands. The effluent from this STW facility drains into St Aubin’s Bay which has led to nitrogen overloading and its current environmental issue: blooms of opportunistic macroalgae smothering the beaches of that bay (e.g., *Ulva* sp.). Other regions around Jersey do not show significant elevation in macroalgae δ^15^N indicating a lack of effluent pollution and therefore, nitrogen loading. This may be the result of oceanic currents, geography and bathymetry of the coastline and/or simply a lack of effluent release to those coastline regions. On the other hand, St Mary’s, IOS, records a complex pattern resulting from an array of unmonitored sewage/effluent sources from all over the island and not from a single source, into a single region (e.g., St Aubin’s Bay, Jersey). Future planning and development of any wastewater treatment plant on St Mary’s requires a thorough investigation of the marine system, especially oceanic currents, tidal effects, marine chemistry, and ecological assessments as standard practice. Case studies on the δ^15^N value of macroalgae from other islands should be conducted to assess the influence and impact of effluent discharge. The use of macroalgae δ^15^N can help identify point source effluent discharges and thus, impact policy changes to mitigate current and future environmental problems/disasters along our coastlines. This study shows that islands are not immune to the environmental issues caused by effluent pollution. This idea was aptly conveyed by John W. Gardner^53^, p. 108 when he wrote, “*we cannot have islands of excellence in a sea of slovenly indifference to standards*”.

## Supplementary Information

Supplementary Information 1.

Supplementary Information 2.

## Data Availability

All data generated or analysed during this study are included in this published article [and its supplementary information files].
