# Supplementary material for: Diffuse and concentrated nitrogen sewage pollution in island environments with differing treatment systems
Source: Sci Rep. 2023 Mar 24;13:4838. doi: 10.1038/s41598-023-32105-6 (PMC10039054; doi:10.1038/s41598-023-32105-6)
Supplement: Supplementary file 2 — Supplementary Information 2. [file 41598_2023_32105_MOESM2_ESM.pdf]

Diffuse and concentrated sewage nitrogen pollution in island environments with differing treatment systems

F. C. Alldred[1], D. R. Gröcke[1], C. Y. Leung[1], L. P. Wright[1], N. Banfield[2]

Page 2: Supplementary Figure S1

Page 3: Supplementary Figure S2

Page 4: Supplementary Figure S3

Page 5: Supplementary Figure S4

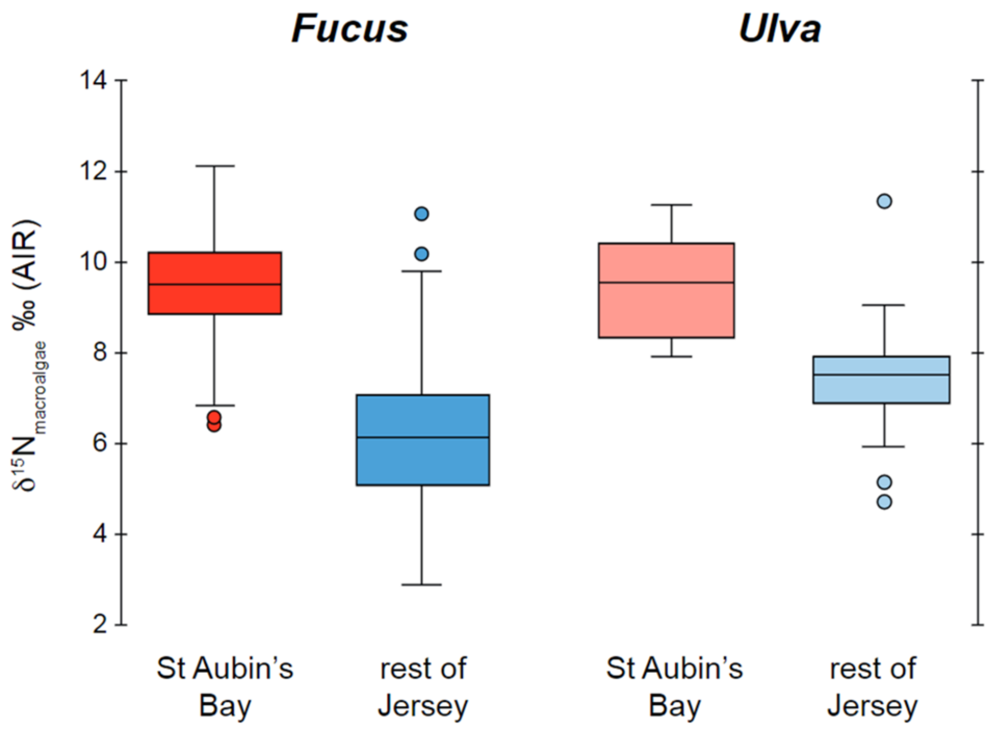

Supplementary Figure S1.

$\delta^{15}N$ box-plot of macroalgae for St Aubin's Bay compared to the rest of Jersey, Channel Islands. Note the significant difference in $\delta^{15}N$ between St Aubin's Bay and Jersey for both species (p value < 0.05). St Aubin's Bay is clearly displaying elevated $\delta^{15}N$ values averaging +9.5 ‰ ± 1.1 ‰ compared to the Jersey average of +6.1 ‰ ± 1.5 ‰ for *Fucus*. *Ulva* averaged +8.3 ‰ ± 1.0 ‰ for St Aubin's Bay, significantly greater than the Jersey average of +6.6 ‰ ± 1.0 ‰.

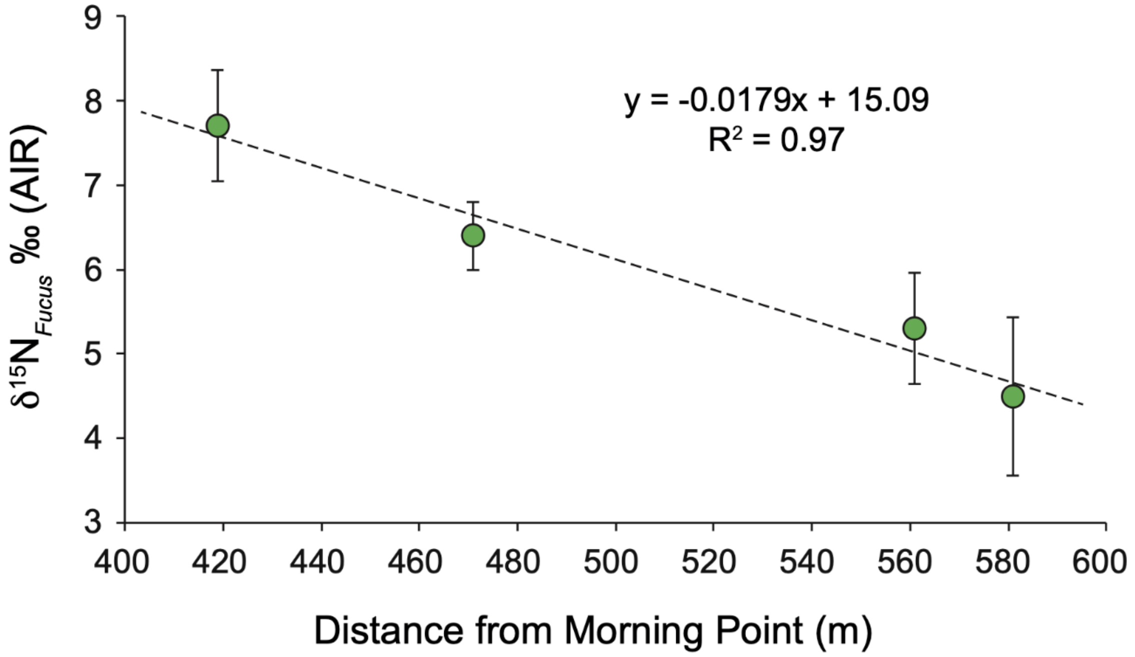

Supplementary Figure S2. $\delta^{15}N$ trend for *Fucus* from Site 42, 44, 45 and 46 in Porth Cressa, St Mary's, Isles of Scilly. Note the strong correlation between elevated $\delta^{15}N$ and distance away from the sewage outflow across a 160 m profile. The decrease rate in *Fucus* $\delta^{15}N$ from the source is ~0.2 ‰ per 10 m.

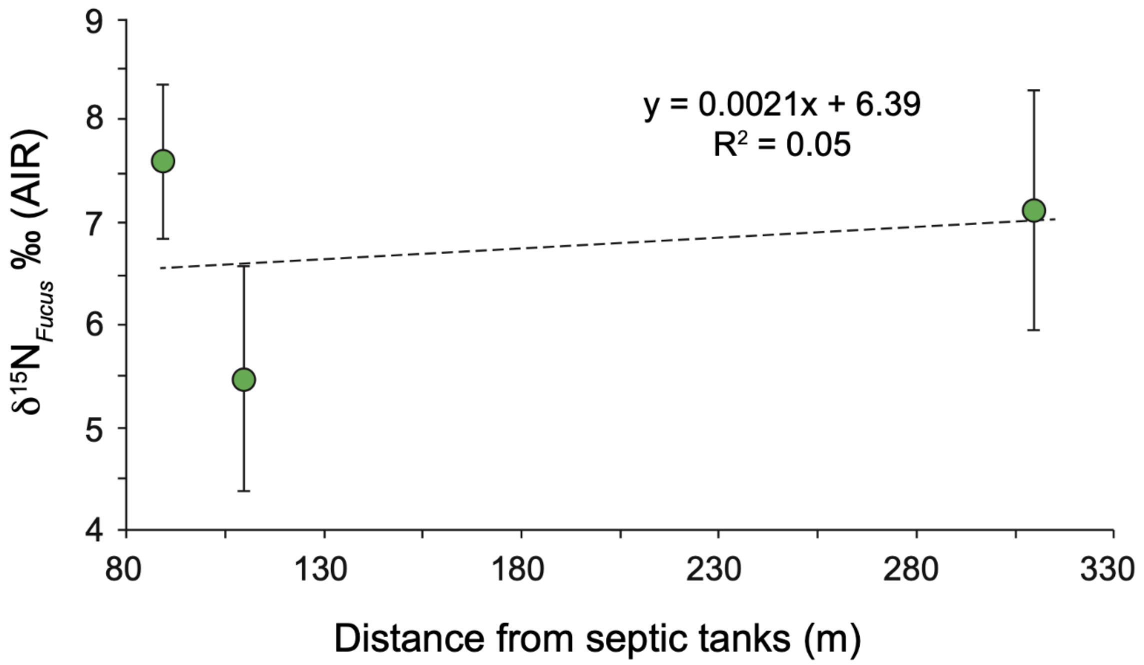

Supplementary Figure S3. $\delta^{15}N$ trend for *Fucus* collected for Site 39, 40 and 41 at Old Town Bay, St Mary's, Isles of Scilly. No correlation ($R^2 = 0.05$) exists between $\delta^{15}N$ and proximity to the three septic tanks located to the east of the bay. Note, the large standard deviations and range in $\delta^{15}N$ values (e.g., > 5.0 ‰) in comparison to Figure S2.

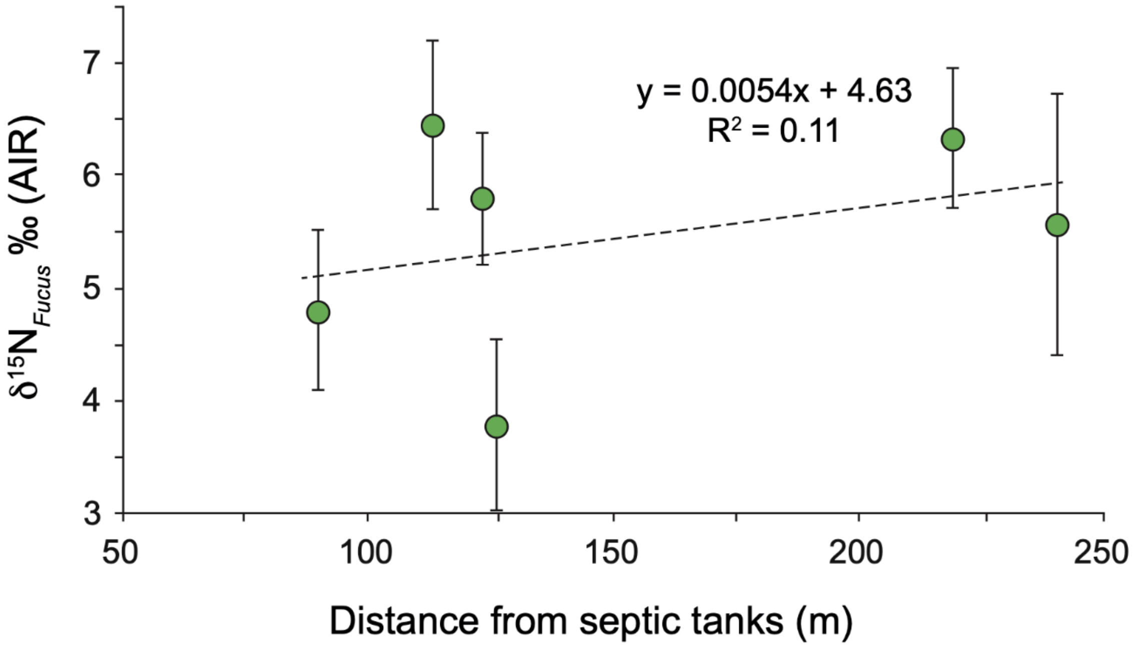

Supplementary Figure S4. $\delta^{15}N$ trend for *Fucus* collected from sites 54–64 in St Mary's Pool, St Mary's, Isles of Scilly. A very weak correlation ($R^2 = 0.11$) between $\delta^{15}N$ and proximity to septic tanks is recorded. In this scenario, macroalgae $\delta^{15}N$ appears to become slightly elevated across the ~200 m profile from the septic tanks. It is possible that the headland at St Mary's Pool is causing effluent to become trapped in the north of the bay and larger $\delta^{15}N$ values are observed further from these septic tanks.
